# A joint NCBI and EMBL-EBI transcript set for clinical genomics and research

**DOI:** 10.1038/s41586-022-04558-8

**Published:** 2022-04-06

**Authors:** Joannella Morales, Shashikant Pujar, Jane E. Loveland, Alex Astashyn, Ruth Bennett, Andrew Berry, Eric Cox, Claire Davidson, Olga Ermolaeva, Catherine M. Farrell, Reham Fatima, Laurent Gil, Tamara Goldfarb, Jose M. Gonzalez, Diana Haddad, Matthew Hardy, Toby Hunt, John Jackson, Vinita S. Joardar, Michael Kay, Vamsi K. Kodali, Kelly M. McGarvey, Aoife McMahon, Jonathan M. Mudge, Daniel N. Murphy, Michael R. Murphy, Bhanu Rajput, Sanjida H. Rangwala, Lillian D. Riddick, Françoise Thibaud-Nissen, Glen Threadgold, Anjana R. Vatsan, Craig Wallin, David Webb, Paul Flicek, Ewan Birney, Kim D. Pruitt, Adam Frankish, Fiona Cunningham, Terence D. Murphy

**Affiliations:** 1 European Molecular Biology Laboratory, European Bioinformatics Institute, Wellcome Genome Campus, Hinxton, UK; 2 National Center for Biotechnology Information, National Library of Medicine, National Institutes of Health, Bethesda, MD USA

**Keywords:** Databases, Genome informatics, Sequence annotation, Standards, Clinical genetics

## Abstract

Comprehensive genome annotation is essential to understand the impact of clinically relevant variants. However, the absence of a standard for clinical reporting and browser display complicates the process of consistent interpretation and reporting. To address these challenges, Ensembl/GENCODE^1^ and RefSeq^2^ launched a joint initiative, the Matched Annotation from NCBI and EMBL-EBI (MANE) collaboration, to converge on human gene and transcript annotation and to jointly define a high-value set of transcripts and corresponding proteins. Here, we describe the MANE transcript sets for use as universal standards for variant reporting and browser display. The MANE Select set identifies a representative transcript for each human protein-coding gene, whereas the MANE Plus Clinical set provides additional transcripts at loci where the Select transcripts alone are not sufficient to report all currently known clinical variants. Each MANE transcript represents an exact match between the exonic sequences of an Ensembl/GENCODE transcript and its counterpart in RefSeq such that the identifiers can be used synonymously. We have now released MANE Select transcripts for 97% of human protein-coding genes, including all American College of Medical Genetics and Genomics Secondary Findings list v3.0 (ref. ^3^) genes. MANE transcripts are accessible from major genome browsers and key resources. Widespread adoption of these transcript sets will increase the consistency of reporting, facilitate the exchange of data regardless of the annotation source and help to streamline clinical interpretation.

## Abstract (web summary)

Matched Annotation from NCBI and EMBL-EBI (MANE) delivers joint transcript sets from Ensembl/GENCODE and RefSeq for standardizing variant reporting in clinical genomics and research.

## Main

For more than 20 years, the RefSeq and Ensembl/GENCODE teams, the two major sources of human genome annotation, have provided high-quality reference gene and transcript sets. These resources are used widely for biological research and discovery, with the choice of set depending on the use case. For instance, RefSeq transcripts are typically used for variant submissions to ClinVar^4^ or for variant descriptions in publications. Conversely, large-scale research projects such as ENCODE^5^, gnomAD^6^, DECIPHER^7^ and GTEx^8^ use the Ensembl/GENCODE set. Although both sets are supported by abundant evidence, the two are not identical owing to differences in curation timing, methodology and interpretation of evidence in data-poor genomic regions. Moreover, sequence differences are present because a few RefSeq transcripts do not perfectly match the reference genome sequence. No simple method has been developed thus far to determine end-to-end equivalence between entire transcripts from the two sources, and navigating these differences can therefore be challenging.

In the clinical context, no accepted standard reference sequence is available for reporting variants. Therefore, individuals or laboratories choose their own transcript, typically according to criteria such as transcript length or creation date. Additionally, resources and tools that are routinely consulted for clinical genomics often differ in their choice of preferred transcript. This can confound data interpretation and may cause errors in variant classification, potentially leading to real clinical harm. These challenges call for a transcript set that can be universally adopted across the clinical and research communities as a biologically informed standard reference for variant reporting to provide consistency across browser displays, resources and tools. Indeed, a 2018 survey^9^ conducted by Ensembl highlighted this need, with the majority of respondents expressing the desire for Ensembl/GENCODE and RefSeq to agree on a primary transcript for each gene. The respondents included approximately 800 individuals, of whom around 35% were healthcare professionals or were working in clinical diagnostics.

## MANE collaboration

To meet community needs, we established the Matched Annotation from NCBI and EMBL-EBI (MANE) collaboration. The initial results of this effort are (1) the MANE Select transcript set, designed to include a single representative transcript for every protein-coding gene for clinical reporting and other applications, and (2) the MANE Plus Clinical set for genes at which the MANE Select transcript alone is inadequate for describing all publicly available pathogenic (P) variants. Key features of the MANE transcripts include end-to-end matching between the exons of Ensembl/GENCODE and RefSeq transcript sequences, perfect alignment to the GRCh38 reference genome assembly^10^ (Discussion) and the use of biologically relevant criteria for transcript selection, such as transcript expression levels and conservation of the coding regions. Together, the two sets eliminate the need to choose between annotations when selecting a default transcript or when reporting variants. Access to the MANE data and detailed documentation on the MANE collaboration is available on the NCBI (https://www.ncbi.nlm.nih.gov/refseq/MANE/) and EMBL-EBI Transcript Archive (Tark; http://tark.ensembl.org/web/mane_project/) websites.

## MANE Select

**Extended Data Fig. 1:**
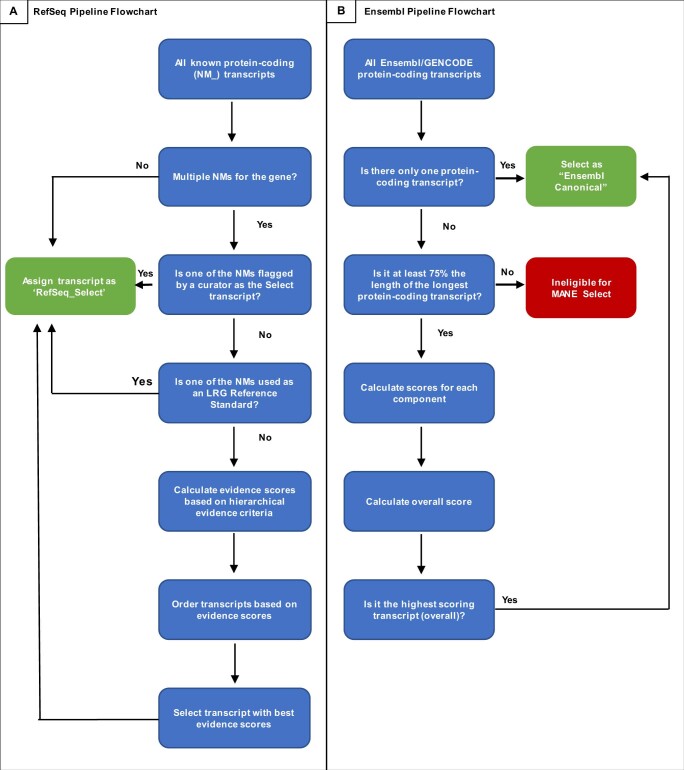
The Select pipelines. **a**, The RefSeq Pipeline picks the Select transcript based on a set of hierarchically scored criteria described in the Methods section and in more detail in Supplementary Method 1. **b**, The Ensembl pipeline assigns Ensembl Canonical to the transcript with the highest score, which is a sum of the component scores for each criteria (e.g. conservation, expression, APPRIS choice, UniProt choice, length). Details are listed in Supplementary Method 1.

To build the MANE Select set, our joint approach involved designing independent pipelines that would each identify representative transcripts for protein-coding genes (Supplementary Methods 1 and Extended Data Fig. 1). We aimed to include all coding exons that are well expressed and show evidence of evolutionary conservation. We then developed a workflow to iteratively compare the pipeline outputs, identify transcript pairs with the same coding sequence (CDS) and exon structure, and standardize the transcript ends.

When using transcripts on GRCh38 available as of May 2018 in our initial comparison of the pipeline outputs, we determined that the Ensembl/GENCODE and RefSeq selections were identical for only 14% of protein-coding genes. In particular, 73% had differences only in the untranslated regions (UTRs), either in the extent of the 5′ or 3′ end or in the choice of UTR exons, and 11% differed in the CDS. For the remaining 2% of genes, we observed other scenarios, such as a missing corresponding transcript in one source. For choices that differed in CDS or UTR exons, we iteratively resolved these differences through pipeline improvements, additional automated data analyses and manual curation following consensus curation guidelines (Supplementary Methods 2). Manual review was aided by quality assurance metrics that flagged discrepancies (Supplementary Table 2).

**Fig. 1:**
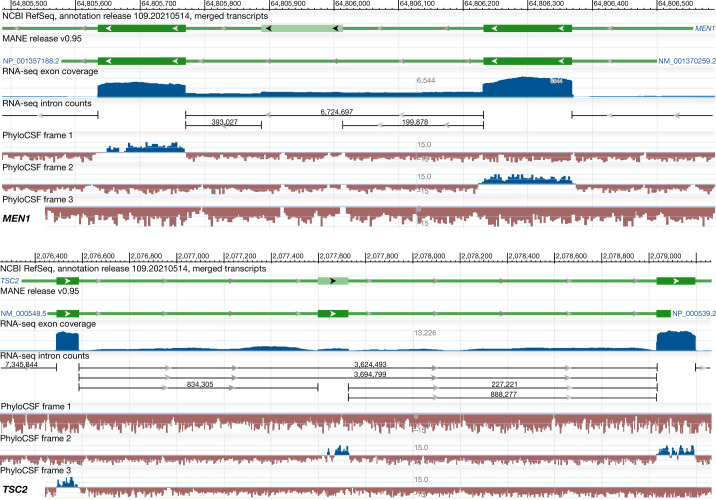
Conservation versus expression when manually curating two high-value clinical genes. Top, gene *MEN1* (HGNC:7010) tracks from NCBI GDV, as described below from top to bottom. Track 1, magnified region of the gene showing a portion of the CDS including an alternatively spliced exon (NCBI annotation release 109.20210514). Track 2, MANE v0.95 track showing the corresponding region of the MANE Select transcript (NM_001370259.2) lacking the alternatively spliced exon. Track 3, RNA-seq exon coverage (aggregate, filtered), with the numbers indicating the peak heights of the graph on a linear scale. Track 4, RNA-seq intron-spanning data from recount3, with horizontal lines depicting introns and numbers above the line indicating the number of reads. Track 5, PhyloCSF tracks. A transcript excluding the alternatively spliced exon was chosen as the MANE Select transcript owing to low expression (tracks 3 and 4) and lack of evolutionary constraint (no positive PhyloCSF signal, as indicated by blue colour) for the alternatively spliced exon. Bottom, gene *TSC2* (HGNC:12363) tracks from GDV, as described below from top to bottom. Track 1, NCBI annotation release 109.20210514 track showing a portion of the coding region. Track 2, MANE v0.95 track showing the corresponding region of the MANE Select transcript (NM_000548.5). Track 3, RNA-seq exon coverage (aggregate, filtered). Track 4, portion of RNA-seq intron-spanning data from recount3. Track 5, PhyloCSF tracks. The MANE Select transcript includes the alternatively spliced protein-coding exon, which, despite its lower expression compared with neighbouring exons, shows evolutionary constraint of the CDS (presence of positive signal in the PhyloCSF track, as indicated by blue colour).

Owing to strong interest from the clinical community, we focused our manual curation efforts on a subset of clinically relevant genes (*n* = 3,803). The clinical relevance of these genes was validated by key clinical partners, including the Transforming Genomic Medicine Initiative (TGMI; http://www.thetgmi.org) and the Clinical Genome Resource (ClinGen)^11^; alternatively, inclusion in repositories such as Genomics England (PanelApp^12^), Gene2Phenotype^13^, OMIM^14^ and ClinVar was used for validation. For genes in the American College of Medical Genetics and Genomics Secondary Findings list (ACMG SF v2.0; ref. ^15^), we reviewed the suitability of the pipeline choice and discussed challenging genes with our clinical partners. Figure 1 illustrates the application of our key criteria, conservation and expression, when choosing a MANE Select transcript for two high-value clinical genes. As mentioned above, our goal was to select transcripts that include well-conserved and well-expressed protein-coding exons. When a coding exon did not meet either criterion (for example, in *MEN1*), a transcript excluding that exon was chosen as the MANE Select transcript. However, a coding exon displaying a signal of conservation was considered for inclusion if it passed our minimum expression threshold, even if this exon was expressed at lower levels than neighbouring exons (for example, in *TSC2*).

**Extended Data Fig. 2:**
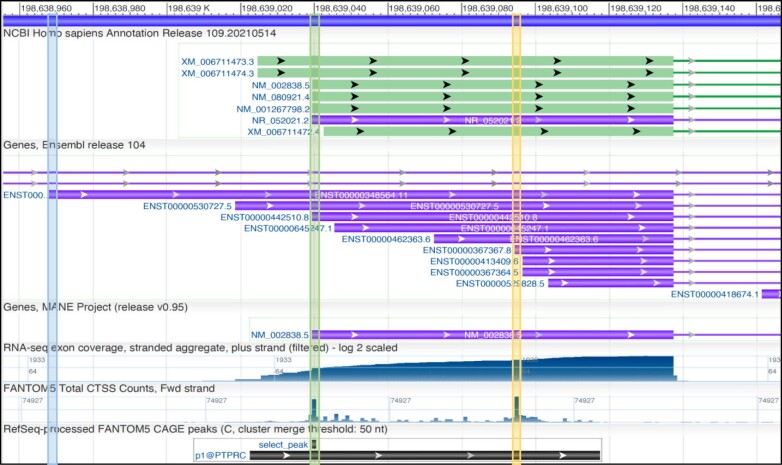
MANE collaboration UTR definition. Graphic display of the 5′ terminal UTR exon of the gene *PTPRC* (HGNC:9666) in NCBI GDV to illustrate how we defined the 5′ end of the transcript. Annotation tracks (top to bottom) show transcripts in RefSeq Annotation Release 109_20210514, transcripts in Ensembl Release 104 and the MANE Select (v0.95) track. The longest 5′ UTR among the RefSeq and Ensembl/GENCODE annotation sets is flagged at the first base with a blue vertical box. The “FANTOM Total CTSS Counts” track displays histograms representing CAGE tag counts at each base position. The strongest CAGE peak (the most abundant start site or the base position with the absolute maximum CAGE tag count) is highlighted with a yellow vertical box. The “RefSeq Processed CAGE” track at the bottom displays the start site (highlighted with a green vertical box) selected by the UTR algorithm. Details of how the UTR algorithm works are covered in the Methods and provided in Supplementary Method 3: UTR algorithm. A similar logic was used to compute polyA clusters and determine the 3′ ends of transcripts.

**Extended Data Fig. 3:**
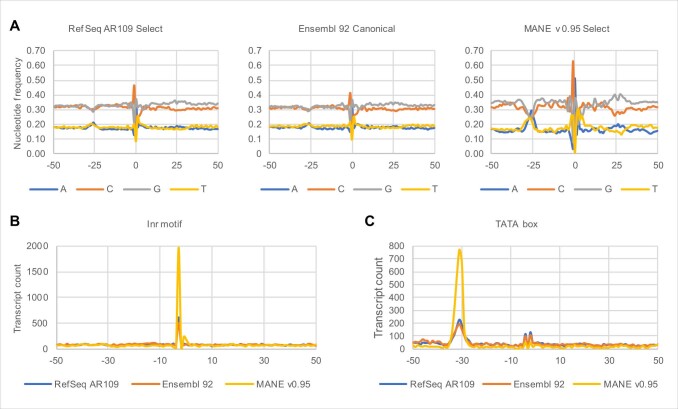
Frequency of TSS signatures in RefSeq, Ensembl, and MANE transcripts. **A**) Frequency of A, C, G, T nucleotides at each position (y-axis) relative to the transcription start site (x-axis). MANE transcripts show an enrichment of C at −1, and purine (A or G) at +1. **B**) Count of transcripts with a best Inr motif (y-axis) placed relative to the TSS (x-axis). The peak of Inr motifs at −3 corresponds to the core CA motif located at −1 to +1. **C**) Count of transcripts with a TATA-box (y-axis) placed relative to the transcription start site (x-axis). The peak of TATA-box motifs at −31 corresponds to the core TATAAA box motif located at −28 to −23 upstream of the TSS. Details of the methods are available in Supplementary Methods 1. Source data

After selecting transcript pairs, with one transcript from each source, we determined the optimal 5′ and 3′ ends on the basis of the supporting evidence. We incorporated high-throughput datasets (described in Methods) to programmatically determine and automatically update the 5′ and 3′ ends of both the RefSeq and Ensembl/GENCODE transcripts, even for some of the pairs that were initially found to be identical. Once updates were completed and perfect identity was achieved, both transcripts in the pair were tagged as MANE Select. Extended Data Fig. 2 illustrates our method to determine the transcription start site (TSS) for the MANE Select transcript of the gene *PTPRC* (HGNC:9666). Similar logic was used to compute poly(A) clusters to determine the 3′ ends of transcripts. Additional details are provided in Supplementary Methods 3. The 5′-end updates of the transcripts resulted in an enrichment for motifs characteristic of eukaryotic transcription initiation^16^, including initiation at purines and the presence of properly positioned TATA box or initiator motifs for a subset of transcripts (Extended Data Fig. 3).

**Extended Data Fig. 4:**
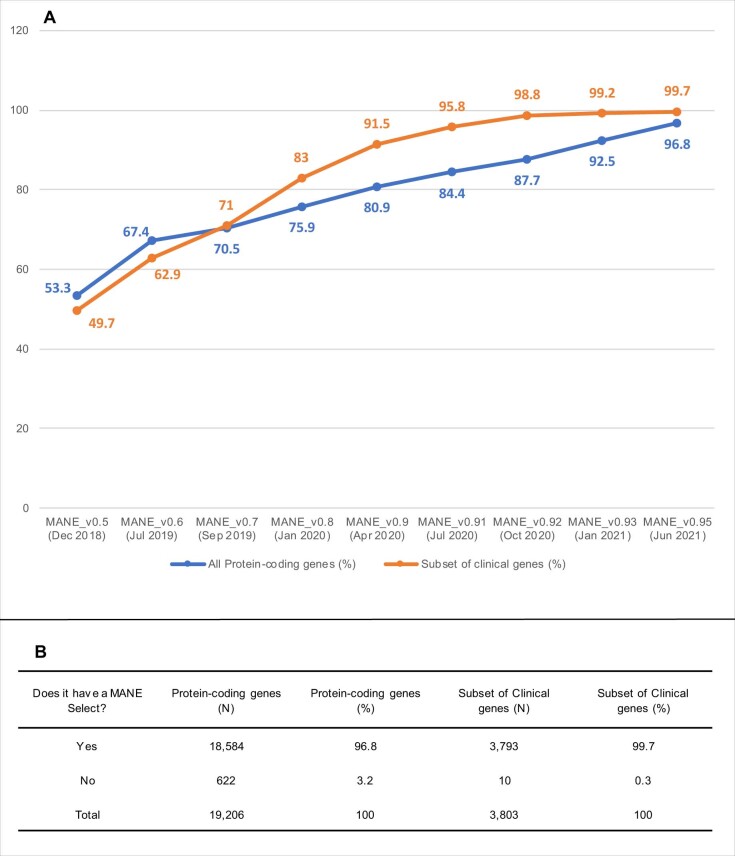
MANE Select coverage over time. (**A**) Graphical display of the percentage of all protein-coding genes (blue) and of the subset of clinical genes (orange) that have a defined MANE Select transcript per each MANE project release over time. (**B**) Number of genes that have a defined MANE Select transcript (MANE v0.95). The list includes 101 genes that will require the MANE Select to be defined using an ALT or PATCH (rather than the GRCh38 Primary Assembly). It does not include an additional set of 345 genes that require review due to conflicting gene types between RefSeq and Ensembl/GENCODE.

By June 2021 (MANE release v0.95), we had defined a MANE Select transcript for 97% (18,584) of protein-coding genes across the genome. This includes all ACMG SF v3.0 genes and more than 99% (3,793 of 3,803) of the subset of disease-associated genes (Extended Data Fig. 4). The outstanding clinical genes include those to be added in the next release (*KLK4*, *TOMT*) or those affected by errors in the GRCh38 chromosome sequences (*ABO*, *FUT3*, *MUC1*, *ORAI1*, *POLR2A*, *SHANK3*) or atypically complex annotation (*PEG10*). We aim to complete the set in early 2022. The vast majority of MANE Select transcripts will be stable. However, we will allow updates on the rare occasion that new data demonstrate, without ambiguity, that the MANE Select transcript requires an update or needs to be replaced with a better transcript.

## MANE Plus Clinical

**Fig. 2:**
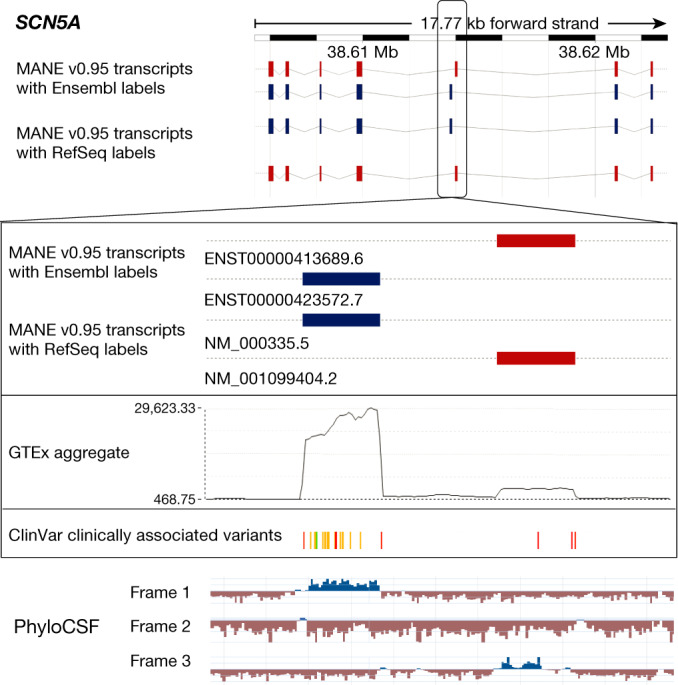
The need for a MANE Plus Clinical transcript for the *SCN5A* (HGNC:10593) gene. Top, Ensembl browser display of the *SCN5A* gene showing MANE Select (blue) and MANE Plus Clinical (red) transcripts (Ensembl/GENCODE on top and RefSeq below) from MANE release v0.95. Bottom, magnified view of the portion of the gene that includes two mutually exclusive exons. The tracks are as described below, from top to bottom. Track 1, MANE v0.95 track showing the upstream MANE Select exon and downstream MANE Plus Clinical exon, shown in blue and red, respectively. Track 2, GTEx aggregate exon coverage (black wiggle plot). Track 3, ClinVar variants described as P or LP, coloured to indicate the type of variant (green, synonymous; yellow, missense; red, stop gained). Track 4, PhyloCSF tracks (one row for each frame) from NCBI GDV, with positive signal shown in blue.

Although the MANE Select set serves as a variant reporting standard for the majority of genes, some clinically relevant genes require more than one transcript to report all known P or likely pathogenic (LP) variants if these variants map to alternatively spliced exons. For cases in which the MANE Select transcript alone is not sufficient to report all known variants, we defined an additional transcript: the MANE Plus Clinical transcript. After consultation with our clinical partners, we have released MANE Plus Clinical transcripts for 55 genes. Figure 2 illustrates the need for a second transcript to report the P and LP variants that map to mutually exclusive exons in the *SCN5A* (HGNC:10593) gene.

## Updates to original transcript datasets

A crucial aspect of the MANE set is the fact that the Ensembl/GENCODE and RefSeq transcripts (and, therefore, proteins) in a MANE pair are identical, and the identifiers can be used interchangeably. To achieve the perfect match, the vast majority of transcripts selected by both pipelines (94% for RefSeq and 94.1% for Ensembl/GENCODE) underwent updates, resulting in version increments (Table 1). This includes some of the transcripts that were identical at the beginning of the project but did not conform to the UTR rules mentioned above. Most of the updates (86% for RefSeq and 88% for Ensembl/GENCODE) were in the UTR. However, a small percentage of transcripts required changes to the CDS (1.8% for RefSeq and 1.5% for Ensembl/GENCODE), which typically involved a change to the location of the start codon. In addition to these updates, new transcripts were created for existing annotations that were incomplete or inconsistent with the MANE criteria (2.4% for RefSeq and 1.6% for Ensembl/GENCODE).

**Table 1:** Updates to RefSeq and Ensembl/GENCODE transcripts.

Type of change	RefSeq	Ensembl/GENCODE
No change (same exons, same CDS)	1,110 (6.0%)	1,094 (5.9%)
5′- or 3′-end change	15,968 (85.9%)	16,336 (87.9%)
New UTR, same CDS	724 (3.9%)	569 (3.1%)
Same exons, CDS changed	328 (1.8%)	285 (1.5%)
New CDS	454 (2.4%)	300 (1.6%)
Total	18,584	18,584

Comparison of RefSeq annotation release 109 (limited to NM_ transcripts) or Ensembl release 92 to MANE release v0.95.

## Comparison with alternative datasets

**Fig. 3:**
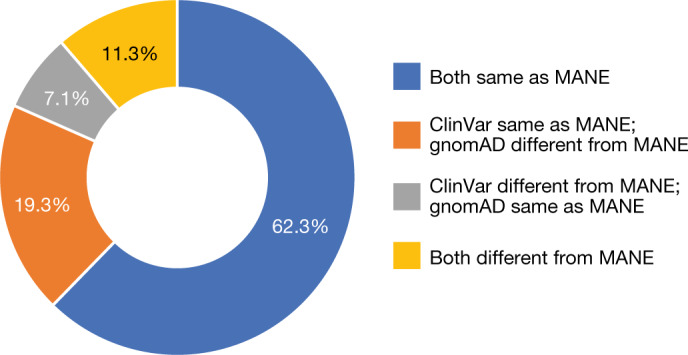
Comparison of the MANE Select dataset with gnomAD and ClinVar. Doughnut chart showing a comparison of MANE Select transcripts with the most frequently used RefSeq transcript accession for variant submission in ClinVar and Ensembl canonical transcripts used for display in the gnomAD v3.1.1 resource. Source data

A goal of the MANE collaboration is to deliver a transcript set that can be widely adopted as a standard for reporting and for display across resources commonly used by the clinical and research communities. To assess the impact of our work, we aimed to quantify the overlap between the MANE Select set and two representative resources, gnomAD and ClinVar, which use Ensembl/GENCODE and RefSeq annotations, respectively. We chose to analyse disease-associated genes and these two resources because they reflect data used in the clinical community, represent orthogonal views of what users are exposed to or are using, and were available with sufficiently broad gene coverage to make the analysis informative. gnomAD shows Ensembl transcripts and could be perceived by users as a recommendation of a particular canonical transcript. The ClinVar submission data indicate which RefSeq transcripts are being used by submitters on the basis of unknown and likely varied criteria. The choice of datasets informed the set of genes included in the analysis. For the subset of manually curated disease-associated genes, we determined whether the canonical transcript in gnomAD (v3.1.1) and the transcript most commonly used for variant submission to ClinVar matched the MANE Select transcript accession. As shown in Fig. 3, the same accession as that for MANE Select was used for 62.3% (*n* = 2,945) of the genes we reviewed. However, different accessions were used in one or both resources for the remaining 37.7% (*n* = 1,779) of genes (7.1% and 19.3% in ClinVar and gnomAD, respectively). This divergence demonstrates the consequence of having no standard transcript set and affirms the aims of our collaboration.

**Extended Data Fig. 5:**
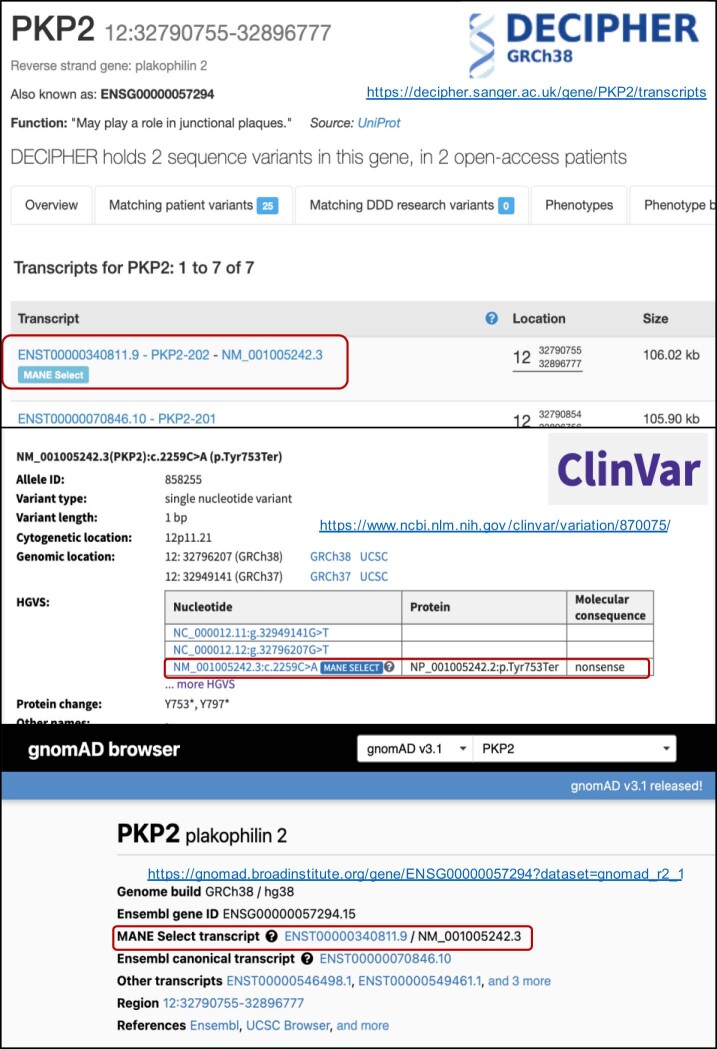
Commonly used resources that have adopted the MANE Select in their browsers and display. Top panel: A screenshot of the gene page of *PKP2* (HGNC:9024) in the DECIPHER database (https://www.deciphergenomics.org/). The transcript table on the gene page shows the MANE Select label with the RefSeq and Ensembl identifiers (marked by a red box). Middle panel: A ClinVar variant display (https://www.ncbi.nlm.nih.gov/clinvar/variation/870075/) page for the gene *PKP2* (allele ID 858255). The HGVS table in this page includes the RefSeq component of the MANE Select (indicated by red box). Bottom panel: A display page from the Genome Aggregation Database gnomAD v3.1. The MANE Select pair, along with the RefSeq and Ensembl identifiers, are displayed at the top of the page (indicated by red box). We note that UniProt, another commonly used resource, will update their browser soon to include flagged MANE Select proteins.

We collaborated with resources such as ExAC/gnomAD, ClinGen, ClinVar, DECIPHER and the Ensembl Variant Effect Predictor (VEP)^17^, all of which had different preferred transcripts, to encourage adoption of the MANE Select set, achieve standardization and ensure consistency. The interfaces of these resources now display the MANE Select transcript (Extended Data Fig. 5). In addition, UniProt is expected to update its browser in the near future to include flagged MANE Select proteins.

## Access and display of MANE data

**Extended Data Fig. 6:**
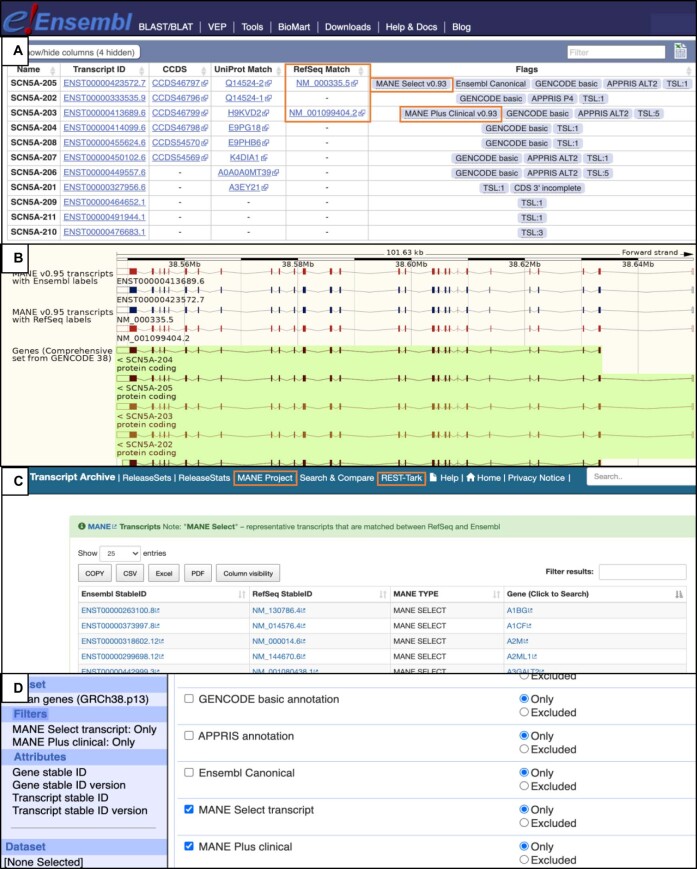
Display of MANE data in Ensembl. (**A**) In Ensembl’s Gene page, the Ensembl/GENCODE transcript(s) in the MANE set is highlighted with the “MANE Select” or “MANE Plus Clinical” flags, visible in the last column of the transcript table. The identical RefSeq transcript is highlighted in the same table, in the column titled “RefSeq Match”. (**B**) Graphical representation is visible in the Location page after configuring the view by adding the custom-made MANE Project track hub (https://ftp.ncbi.nlm.nih.gov/refseq/MANE/trackhub/hub.txt). (**C**) The list of MANE transcripts can be accessed and downloaded from Ensembl’s Transcript Archive (Tark) MANE Project page (http://tark.ensembl.org/web/manelist) and programmatically using APIs available in the REST API page (http://tark.ensembl.org/api/#!/transcript/transcript_manelist_list), or Ensembl’s REST API e.g. https://rest.ensembl.org/overlap/id/ENSG00000128573?feature=mane;content-type=text/xml. (**D**) MANE data can also be downloaded from Ensembl BioMart (https://www.ensembl.org/biomart/martview/c24cb3213fe65da552fcb8b755c2910c) by choosing the ‘Human Genes (GRCh38.p13) dataset and the ‘MANE transcripts’ filter.

**Extended Data Fig. 7:**
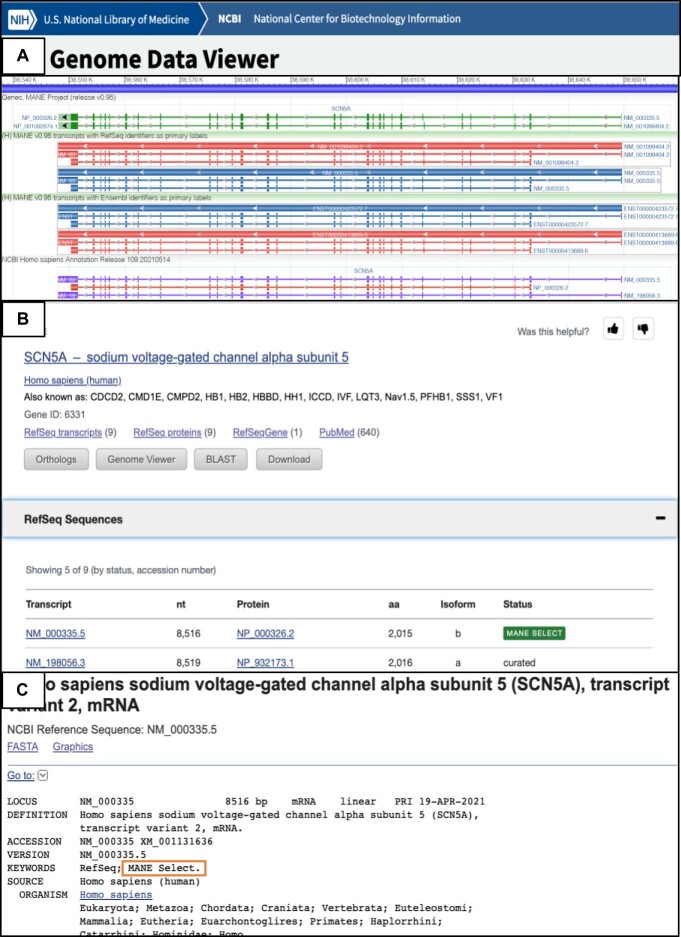
Access to MANE data in NCBI resources. (**A)** Genome Data Viewer (GDV). The MANE track (green, at the top) shows RefSeq transcripts assigned as MANE Select and MANE Plus Clinical for the gene *SCN5A* (HGNC:10593). The middle section shows RefSeq and Ensembl identifiers included in the MANE sets, available by adding the MANE track hub using the ‘Configure Track Hubs’ menu. The bottom section shows a portion of RefSeq annotation release 109.20210514. (**B**) The gene search results page (shown here for the gene *SCN5A*), reached by searching for any human protein-coding gene in https://www.ncbi.nlm.nih.gov/gene/, flags the MANE Select in the expanded transcript list. (**C**) A portion of the transcript record of NM_000335.5, the MANE Select for *SCN5A*. The MANE Select tag (boxed) is included in the ‘KEYWORDS’ section. The keyword can be used in Nucleotide and Protein database queries to extract a list of MANE Select transcripts. For example: PALM[gene] AND MANE Select[keyword]. The entire list of MANE Select transcripts can be obtained using the Entrez query “Homo sapiens[organism] AND MANE_select[keyword]”. MANE data can also be parsed from the annotation files available in the NCBI RefSeq FTP page (https://ftp.ncbi.nlm.nih.gov/refseq/H_sapiens/annotation/annotation_releases/109.20210514/GCF_000001405.39_GRCh38.p13/) using the “MANE Select” tag attribute (tag=MANE Select in GFF3, or tag ”MANE Select'' in GTF), in the rows associated with the mRNA, CDS and exon features. In addition, column 9 also contains the matching Ensembl transcript identifier as an external database reference (Dbxref). Rows in the annotation files associated with the CDS feature contain the MANE Select tag, along with the matching Ensembl protein identifier.

**Extended Data Fig. 8:**
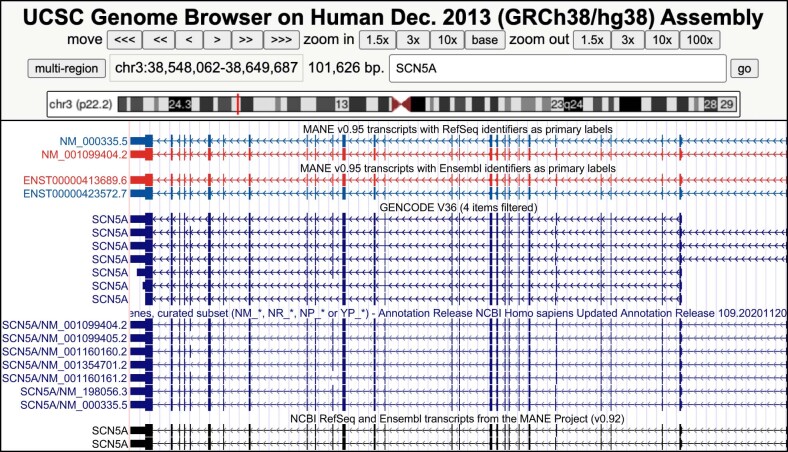
Access to MANE Data in UCSC browser. The MANE data are accessible in UCSC’s Genome Browser as a data track in the Genes and Gene Predictions section (bottom of figure). MANE data can also be viewed in this browser by adding the track hub (https://ftp.ncbi.nlm.nih.gov/refseq/MANE/trackhub/hub.txt), which displays the RefSeq and Ensembl identifiers of the MANE Select separately (top of figure), as shown in this display of the *SCN5A* (HGNC:10593).

All data produced by the MANE collaboration are freely accessible in genome browsers, by bulk download and programmatically (see links in Extended Data Table 1). A complete list of MANE transcripts with RefSeq and Ensembl identifiers in the latest MANE release﻿ (v0.95) is available in the MANE.GRCh38.v0.95.summary.txt.gz file on the FTP site (https://ftp.ncbi.nlm.nih.gov/refseq/MANE/MANE_human/release_0.95/) and Tark (http://tark.ensembl.org/web/manelist). As shown in Extended Data Fig. 6, the Ensembl browser displays MANE data using a custom-made track hub and labels the MANE transcripts in the transcript table within the gene-specific pages. The NCBI Genome Data Viewer (GDV)^18^ allows display of tracks for each MANE release and includes MANE tags in the RefSeq annotation (Extended Data Fig. 7). In addition, the University of California, Santa Cruz (UCSC) Genome Browser^19^ allows selection of a MANE data track in the Genes and Gene Predictions section and exploration of the data in the Table Browser tool (Extended Data Fig. 8).

## Discussion

**Extended Data Fig. 9:**
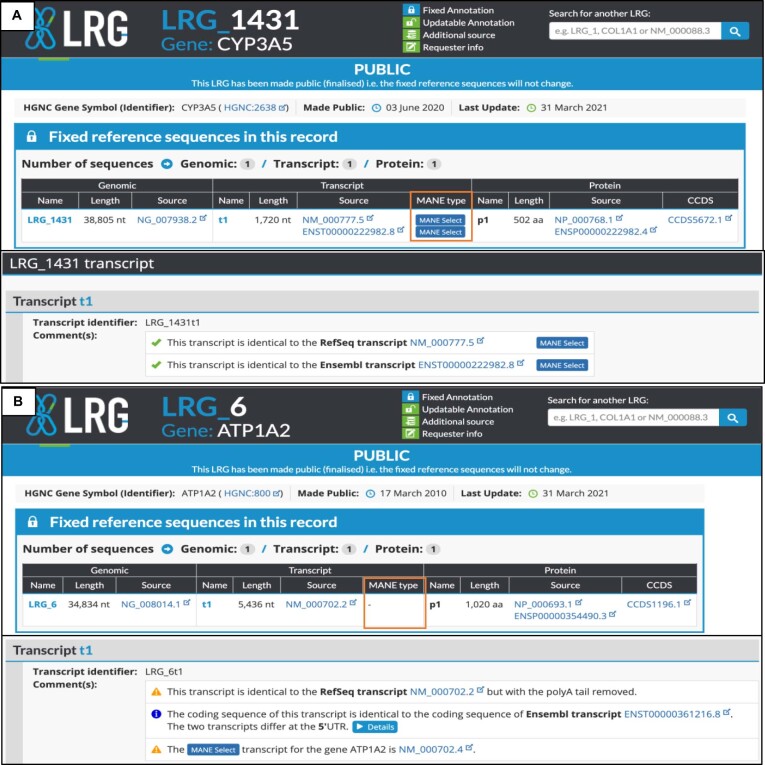
MANE transcript display in LRG records. Screenshots of the LRG records for the genes *CYP3A5* (HGNC:2638) (http://ftp.ebi.ac.uk/pub/databases/lrgex/LRG_1431.xml) and *ATP1A2* (HGNC:800) (http://ftp.ebi.ac.uk/pub/databases/lrgex/LRG_6.xml) displaying MANE transcript annotations. As illustrated in this figure, if the LRG and MANE Select transcripts are identical (Panel A, LRG_1431 for *CYP3A5*), the MANE Select flag is displayed in the Fixed Reference Sequence and Transcript sections of the LRG. In the event that the LRG transcript is not the MANE Select (Panel B, LRG_6 for *ATP1A2*), there will be no flag in the Fixed reference section but the MANE Select transcript will be listed in the Transcript section for the user’s information.

RefSeq and Ensembl/GENCODE have collaborated in the past to converge on annotation and provide joint, high-quality, evidence-based reference sets. We initiated the Consensus Coding Sequence (CCDS)^20^ project in 2005 to provide transcript coding regions consistently annotated by the two groups. In 2008, we established the Locus Reference Genomic (LRG)^21^ project to provide stable reference sequences to report clinical variants. The MANE project goes beyond these collaborations in scope and content. It is not limited to coding regions, as in CCDS, but provides end-to-end matches between transcripts from the two sources. MANE is an improvement over LRG because, in addition to covering all protein-coding genes rather than a limited set of clinical genes, it provides transcript annotations that perfectly match the reference assembly. This is vital to reduce errors, considering that diagnostic pipelines now use whole-exome sequencing or whole-genome sequencing or will implement these methods in the near future. Therefore, NCBI and EMBL-EBI leaders of the LRG project decided to keep the LRG webpage and existing data available but have stopped expanding the LRG set. We recommend using the MANE transcript sets over those of LRG as a reference standard for clinical reporting. Existing LRG accessions now incorporate MANE transcript annotation (Extended Data Fig. 9) and will continue to be supported. Moreover, the Human Genome Variant Society (HGVS)^22^ now includes a recommendation to use MANE transcripts in its general and reference sequence guidelines.

### Caveats and limitations

Selection of one transcript does not imply that the rich biology of the human genome can be reduced to one transcript at each locus, nor does it mean that transcripts not included in the MANE set are inferior or can be ignored. Even though the MANE set drives standardization for browser display and clinical reporting, we are not suggesting that only MANE transcripts be considered when analysing variants of potential clinical significance. For example, some disease mechanisms involve regulating expression in a tissue-specific manner or during a particular stage of development. This level of specificity and transcript diversity is not within the scope of the MANE Select set. Furthermore, when generating the MANE Plus Clinical set, we considered only P or LP exonic variants reported in ClinVar or other public resources. Given that not all laboratories make their variants freely accessible, our Plus Clinical set is a work in progress. We expect the set to increase as new variants are discovered and reported in public archives. Although this work has been driven by our annotation expertise, feedback from the community is encouraged. We will consider additional transcripts of clinical interest after consulting clinical experts. Enquiries about existing MANE transcripts and addition of new transcripts may be sent to mane-help@ncbi.nlm.nih.gov or mane-help@ebi.ac.uk.

The MANE sets are currently limited to protein-coding genes. We anticipate including well-supported non-coding genes in the future, particularly those with clinical relevance. In addition, a small percentage of protein-coding genes cannot currently be matched between RefSeq and Ensembl owing to errors in the GRCh38 primary reference assembly. We are collaborating with the Genome Reference Consortium (GRC) to generate patch sequences that correct errors or improve the assembly. GRC has indefinitely postponed the release of GRCh39; therefore, some protein-coding genes in MANE will have annotation on a patch. Additionally, mitochondrial genes and genes that undergo ribosomal slippage, such as *PEG10*, are presently not included in the MANE sets. However, we intend to include them in the future.

The MANE transcript sets are based on GRCh38 by design; thus, we plan to keep MANE matched only to GRCh38 for the foreseeable future to provide a unified stable clinical reporting standard. Most users are well served by a single reference genome assembly used uniformly across different resources. The most recent research data, analysis and annotation are available exclusively on GRCh38, which is supported in key clinical resources and tools such as gnomAD, ClinVar and DECIPHER. Accordingly, RefSeq and GENCODE will continue using GRCh38 as the primary annotation reference for years to come. However, we recognize that many clinical laboratories will continue to use GRCh37 and that there is interest in new complete or nearly complete genome assemblies representing additional population diversity^23^. Thus, RefSeq and Ensembl/GENCODE will develop further resources and tools to enable future pan-genomes and variation in other assemblies to be interpreted relative to MANE transcripts. For example, the RefSeq annotation of GRCh37, updated in March 2022, is available with markup for RefSeq Select transcripts, including those mapped to GRCh37 from MANE v0.95. A comparison of MANE transcripts to Ensembl/GENCODE annotation on GRCh37 is available at http://tark.ensembl.org/web/mane_GRCh37_list/. Mappings of MANE annotation from GRCh38 will be available on additional human assemblies in the future from both RefSeq and Ensembl/GENCODE. However, because MANE transcripts are planned to be generated only on GRCh38, those mapped to other assemblies may have sequence differences (for example, for 5% of genes in GRCh37), which need to be accounted for when generating HGVS expressions. We therefore recommend broad adoption of GRCh38 in the clinical community to take full advantage of MANE, improve consistency in variant identification and promote the exchange of clinical variant data. Failure to transition could cause discordance in variant identification^24^, making variant interpretation vulnerable to outdated or incomplete genome annotation and severely limiting the exchange of clinical variant reports.

### Future plans

We expect to finalize the MANE Select set in early 2022 (or finish as close to 100% of genes as possible given the limitations mentioned above) and to iteratively extend the MANE Plus Clinical set as new P variants are discovered. We are working with UniProt to align its set with MANE Select to provide access to a wealth of protein-based annotation in a consistent manner. Genes not currently in the MANE set include those for which the annotation differs between Ensembl/GENCODE and RefSeq owing to locus complexity and lack of evidence. In addition, genes needing genome patches and those annotated in only one of the two sets will be manually reviewed.

In the long term, we aim to produce a new set to include additional high-value transcripts, including those for the non-coding genome, such as transcripts that carry exclusive, well-conserved exons that utilize alternative promoters or that have different termini. We will work on this set once we have mature workflows to integrate long transcriptomic data and data arising from rapid technical advances in the wider transcriptomics and proteomics fields. We are also considering the development of a set to label genes and transcripts relevant for human diseases. As a starting point, we plan to use the sets of genes defined by groups that are actively assessing gene–disease validity, such as the global Gene Curation Coalition (https://thegencc.org/).

In summary, as a result of our efforts to converge on the annotation of human protein-coding genes, our collaboration initiative between RefSeq and Ensembl/GENCODE delivers a joint transcript set to standardize clinical genomics and research. This set of one transcript per gene can be used as a default for tools and resources and as a reference set for clinical reporting and research. Universal adoption of this high-value set will promote consistency in reporting, limit clinical harm caused by errors in interpretation, increase the bidirectional exchange of data and help drive improvements in human health and diagnostics.

## Methods

### MANE Select workflow

To produce the MANE Select set, (1) both annotation groups developed pipelines to choose a representative transcript; (2) the two pipeline choices were compared; (3) the matched choices were updated to adjust the ends; and (4) when the two pipeline choices did not match, they were binned into multiple categories (Supplementary Table 1) to be resolved by pipeline refinements or manual review (Supplementary Methods 2). Although the two pipelines are described in detail in Supplementary Methods 1 and Extended Data Fig. 1, the key features are outlined here. The Ensembl pipeline takes into account evidence of functional potential, including transcript expression levels (Intropolis^25^ and recount3; ref. ^26^) and evolutionary constraint of the coding region (Phylogenetic Codon Substitution Frequencies, PhyloCSF^27^). Other factors are CDS length and concordance with the APPRIS^28^ principal isoform and the UniProt/Swiss-Prot^29^ canonical isoform. The pipeline assigns a score for each component from which a composite score is derived. The transcript with the highest composite score is selected as the Ensembl choice, although some length exceptions apply. The RefSeq Select pipeline uses a hierarchical list of parameters, with prior use in clinical reporting and conservation of the coding region (PhyloCSF) at the top. Each parameter is assigned a binary score, and the RefSeq Select transcript is chosen on the basis of a composite score reflecting the ranked choice of the individual parameters.

### Defining UTRs

To standardize the 5′ and 3′ ends of the transcripts, we used high-throughput cap analysis of gene expression (CAGE) data from the FANTOM consortium^30^ and poly(A)-seq data from multiple studies^31–37^, respectively. For the 5′ ends, we imported the CTSS TotalCounts data included for 2,006 runs of CAGE sequence data on the HelicoScope platform from 1,829 distinct samples mapped to the GRCh38 assembly (BioProject, PRJDB1099). The FANTOM data were reprocessed to combine CAGE clusters found in close proximity (within 50 nucleotides of each other) on the same strand and to re-analyse the TotalCounts data in the region of each merged cluster to find the maximum peak. The TSS was then recalculated to be the 5′-most peak in the merged cluster with a signal of at least 50% of the maximum peak. This criterion is referred to as the ‘longest strong’ rule. The goal of the reprocessing is to determine a frequently used TSS that is representative of the overall data rather than that with the absolute maximum tag counts. In this way, we maximize the coverage of commonly observed 5′-UTR bases (and any sequence- or structure-based features they contain). The reprocessed CAGE tracks are available from NCBI GDV as RefSeq-processed FANTOM CAGE peaks tracks. To update the transcripts to the calculated longest strong TSS, we used an automated process that identified CAGE clusters overlapping the first exons of transcripts or those within 500 nucleotides of the first nucleotide. Alternatively, we updated them manually when the genes required additional review. We followed a similar logic for the 3′ end and poly(A)-seq clusters (Supplementary Methods 3).

### Comparison of transcript ends with genomic TSS signatures

We scanned the genomic sequence for the following TSS signatures: (1) enrichment of purines (A or G), which is characteristic of RNA polymerase II transcription initiation, and (2) TATA box motifs at about −30 and initiator^38^ motifs at −1 relative to the TSS. We performed a comparison with two datasets, transcripts at the beginning of this project as well as predating bulk CAGE-based transcripts and those in the current MANE set. We used HOMER^39^ to analyse nucleotide frequencies, the FIMO^40^ tool from the MEME suite to scan for motifs using a position weight matrix (PWM) from JASPAR^41^ for analysis of TATA boxes and a PWM from ref. ^38^ for analysis of the initiator motif. The 200-nucleotide sequence centred on each TSS was scanned using FIMO, and the position of the highest scoring match to the PWM was recorded using a *P*-value threshold of 0.01. Additional details are provided in Supplementary Methods 3 and Extended Data Fig. 3.

### MANE Plus Clinical workflow

The starting point for the MANE Plus Clinical set was the list of known P and LP variants available in the ClinVar 20200513 release. All P and LP variants were considered, regardless of their review status (‘star’ designation). We identified transcripts that contained conserved coding exons not represented in the MANE Select set and that overlapped these P or LP variants. This set of additional transcripts was manually reviewed to ensure the same high degree of quality as for the transcripts in the MANE Select set.

### RefSeq and Ensembl/GENCODE transcript updates

The annotation comparison logic used for the MANE workflow (Supplementary Methods 4) was adapted to compare transcripts from the early RefSeq and Ensembl/GENCODE annotation sets with those of the most recent MANE release. The first comparison was carried out using the human RefSeq 109 and Ensembl 92 annotation sets. For each MANE Select transcript, the comparison dataset was checked for transcript and CDS annotations that were completely identical; differed only in the extent of the 5′ and 3′ UTRs; differed in the CDS but had the same transcript splice pattern, indicating a change in start codon; or cases in which a transcript lacked an equivalent splice pattern. The comparisons were performed independently of transcript identifiers; in some cases, a transcript was indicated as ‘new’ when it was an update of an existing transcript but exons were added or removed. The comparisons did not consider sequence changes or the removal of poly(A) tails from some RefSeq transcripts, which resulted in additional updates.

### Reporting summary

Further information on research design is available in the Nature Research Reporting Summary linked to this paper.

## Online content

Any methods, additional references, Nature Research reporting summaries, source data, extended data, supplementary information, acknowledgements, peer review information; details of author contributions and competing interests; and statements of data and code availability are available at 10.1038/s41586-022-04558-8.

### Supplementary information

Supplementary Methods This file provides additional information on the methodology used to produce the MANE transcript sets.

Reporting Summary

Supplementary Table 1 This file provides a list of quality assurance criteria analysed to determine the suitability of a transcript for inclusion in the MANE Select set or for automatic updates to the 5′ and 3′ UTRs of transcripts.

### Source data

Source Data Fig. 3

Source Data Extended Data Fig. 3

## Figures and Tables

**Extended Data Table 1:**
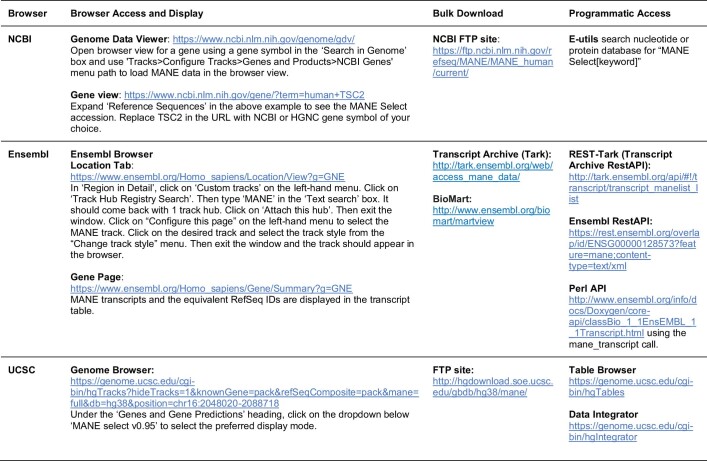
Links to access MANE data from Ensembl, NCBI and UCSC

Examples are provided for browser access in each genome browser. Columns 2 and 3 have links for bulk download of the data and for programmatic querying.

## Data Availability

The datasets generated during the current study are available on the NCBI FTP site (https://ftp.ncbi.nlm.nih.gov/refseq/MANE/MANE_human/) and the Tark webpage (http://tark.ensembl.org/web/mane_project/). Source data are provided with this paper. The datasets analysed during the current study can be accessed using the following resources. All Ensembl/GENCODE annotation builds used in the comparison of RefSeq and Ensembl/GENCODE transcripts for determination of transcript matches in the MANE analysis are available in the release 96–105 directories on the Ensembl FTP site (http://ftp.ensembl.org/pub/release-105/gtf/homo_sapiens/Homo_sapiens.GRCh38.105.gtf.gz). All RefSeq annotation builds used in the comparison of RefSeq and Ensembl/GENCODE transcripts for determination of transcript matches in the MANE analysis are available at https://ftp.ncbi.nlm.nih.gov/genomes/refseq/vertebrate_mammalian/Homo_sapiens/annotation_releases/. The Ensembl canonical transcripts used for the comparison of gnomAD versus ClinVar versus MANE were from Ensembl release 103. These can be accessed using the Ensembl Perl API for release 103 with the following call on the gene: http://www.ensembl.org/info/docs/Doxygen/core-api/classBio_1_1EnsEMBL_1_1Gene.html. Alternatively, the same data are available through the Ensembl REST API by using the following lookup endpoint: https://jan2020.rest.ensembl.org/documentation/info/lookup. The aggregated CTSS TotalCounts CAGE data and the CAGE clusters as computed by the FANTOM consortium were imported from http://fantom.gsc.riken.jp/5/datafiles/reprocessed/hg38_latest/extra/CAGE_peaks/hg38_fair+new_CAGE_peaks_phase1and2.bed.gz and https://fantom.gsc.riken.jp/5/datahub/hg38/reads/. The poly(A)-seq data used to generate the poly(A) clusters and to determine the poly(A) sites were from multiple studies listed in refs. ^31–37^. The data are available in study accessions SRP041182, SRP003483, SRP007359 and SRP133500 in the NCBI Sequence Read Archive (SRA; https://www.ncbi.nlm.nih.gov/sra) and at PolyASite 2.0 (https://www.polyasite.unibas.ch/). APPRIS data are available at https://appris.bioinfo.cnio.es/#/downloads, which is updated for every Ensembl/GENCODE release. These data are based on Ensembl releases 95–104. The PhyloCSF data used to identify conserved sequences were imported from https://data.broadinstitute.org/compbio1/PhyloCSFtracks/. Intron support data from Snaptron/recount3 were imported from http://snaptron.cs.jhu.edu/data/. Source data are provided with this paper.

## Code availability

The analysis code used for this study is largely integral to the RefSeq and Ensembl/GENCODE curation databases and was not designed for use in isolation or with other annotation datasets. The most critical aspect of the analysis was the tens of thousands of working hours spent in curator review to produce the final dataset. HOMER v4.11 is available at http://homer.ucsd.edu/homer/. FIMO v5.3.2 is available at https://meme-suite.org/meme/meme_5.3.2/doc/fimo.html. HISAT 2.2.1 is available at http://daehwankimlab.github.io/hisat2/.
