# Supplementary material for: A joint NCBI and EMBL-EBI transcript set for clinical genomics and research
Source: Nature. 2022 Apr 6;604(7905):310–5. doi: 10.1038/s41586-022-04558-8 (PMC9007741; doi:10.1038/s41586-022-04558-8)
Supplement: Supplementary file 1 — This file provides additional information on the methodology used to produce the MANE transcript sets. [file 41586_2022_4558_MOESM1_ESM.pdf]

nature portfolio

https://doi.org/10.1038/s41586-022-04558-8

Supplementary information

A joint NCBI and EMBL-EBI transcript set for clinical genomics and research

In the format provided by the authors and unedited

Supplementary Materials

Supplementary Method 1: Description of “Select” pipelines

RefSeq Select pipeline

The RefSeq Select pipeline picks the Select transcript based on a set of hierarchically scored criteria. The pipeline is built for multiple taxa, which will be represented in the RefSeq Select set in the future, with some criteria (such as used as a Reference Standard on public LRG) being specific to the human set. Additionally, as shown in Extended Data Figure 1, only known/curated (NM_) RefSeqs are considered for the human RefSeq Select set. The known RefSeqs are then assigned a binary score for a set of hierarchical criteria. If a gene has a single known RefSeq, it is automatically assigned as the RefSeq Select. When a gene has multiple known RefSeqs, the transcript with the best composite score, derived from scores based on the individual criteria, is chosen as the RefSeq Select.

The pipeline is run on a weekly basis to include new curated RefSeqs. RefSeq Select transcripts chosen by the automated pipeline can be overridden by RefSeq curators after review of the evidence according to a set of curation guidelines. Once a RefSeq acquires the 'MANE Select' tag after a public MANE release, it is excluded from future changes by the RefSeq Select pipeline.

A brief description of the evidence criteria is provided below and summarized in Extended Data Figure 1:

1. Curated Select pick: A transcript designated as RefSeq Select by a curator overrides all other criteria.
2. Prior use as a clinical standard: A RefSeq that is in use as the 'Reference Standard' in an LRG record (https://www.lrg-sequence.org/index.html).
3. Curated (NM_, NP_, NR_) versus non-curated (XM_, XP_, XR_) RefSeqs: In the human and mouse RefSeq Select set, curated (or 'known') RefSeqs (see https://www.ncbi.nlm.nih.gov/refseq/about/) are chosen by default.
4. Accession type (NM_/XM_ versus NR_/XR_): For protein-coding genes, preference is given to coding (NM_, XM_) over non-coding (NR_, XR_) RefSeqs. The MANE Select set only includes NM_ transcripts.
5. PhyloCSF positive score: Evolutionary constraint of the coding region is calculated based on PhyloCSF data. PhyloCSF scores are calculated based on codon substitution frequencies using reference genomes in a set of organisms representing a range of taxonomic groups. Positive PhyloCSF scores indicate evolutionary constraint of coding region (CDS). Preference is given to transcripts with maximum positive scoring bases in the CDS, treating transcripts with similar scores (within 90-bp of the maximum) as equivalent. The union of PhyloCSF data computed from the 100-vertebrate and 58-mammal alignment datasets are used, such that a base position in the CDS with a positive PhyloCSF score in either dataset is considered as being under positive selection, and the total bases are summed to determine the total score for each transcript.
6. Max. expression (0-0.5): A composite expression score (with a score of 0 indicating highest expression) is calculated for each transcript based on 'read scores' (number of short-read RNA-seq sequences spanning the intron, also referred to as 'split reads') of

individual introns, which are based on the combination of short-read RNA-seq studies used in the RefSeq annotation and available long-read data. The score penalizes introns that are under-represented compared to their neighbors to compensate for 3'-to-5' bias in RNA-seq data and favors more splices as a proxy for favoring full-length transcripts. Transcripts with expression scores in the range 0-0.5 are considered to have similar enough expression to be treated equally and defer to a lower ranking criteria.

7. Protein match to SwissProt canonical: The encoded protein matches the 'canonical isoform' in the UniProt/SwissProt set.
8. CAGE expression: The transcript is using a promoter with a CAGE score within 70% of the strongest CAGE cluster associated with any RefSeq transcript (known or model) for the gene.
9. PhyloCSF negative score: The transcript with the least negative score is preferred over other transcripts. Note: PhyloCSF has limitations in certain situations, for example very short exons. Such cases are deferred to manual review.
10. Max protein length: The transcript encoding the longest protein among competing transcripts with the same evidence scores for criteria 1 through 9.
11. Min. expression: This criterion eliminates the transcript with the lowest expression score among competing transcripts with the same evidence scores for criteria 1 through 10.
12. Oldest NM: This last criterion picks the oldest RefSeq (smallest accession number) among competing transcripts with the same evidence scores for criteria 1 through 11.

Ensembl Pipeline

The Ensembl pipeline follows several steps (summarized in Extended Data Figure 1) to select the transcript with the highest score, which is a sum of the component scores based on the following:

1. Conservation:

    - PhyloCSF is a comparative genomics method to calculate the likelihood that a given sequence is evolving as CDS. It is therefore a proxy for conservation. The number of bases that have a positive value in the PhyloCSF data for each exon of a transcript are counted and divided by the length of each coding exon. This score is then normalised with respect to all transcripts at the locus to produce the "PhyloCSF score".

2. Expression, using two types of data:

    - RNA-seq supported intron data (for example, Intropolis in human or Recount 3 (http://rna.recount.bio/) are used to calculate the expression of each transcript. Supporting reads for each intron (RNAseq reads that span the intron) are summed to obtain an overall intron count. The total counts are divided by the number of introns. This value is then normalised with respect to all transcripts at the locus to produce the "Intropolis score". Intropolis is a compilation of exon-exon junctions and the expression, overall and tissue-specific, associated with these junctions.

    - CAGE (Cap Analysis of Gene Expression) data are used to calculate the expression of transcripts that start at different promoters. This deep sequencing technique measures the 5' ends seen in the sample. The algorithm sums the

CAGE read counts that overlap the 5'UTR of each transcript. This value is then normalised with respect to all transcripts at the locus to produce the "CAGE score".

3. Concordance with the APPRIS Principal (P1) CDS isoform:

    ○ The APPRIS database uses functional annotation and cross-species conservation to select a principal coding isoform. The algorithm assigns a score to all transcripts at the locus whose CDS is identical to the APPRIS' Principal (P1) CDS isoform. This is the "APPRIS score".

4. Concordance with the UniProt canonical protein isoform:

    ○ UniProt has defined a canonical protein isoform for all human protein-coding genes. The algorithm assigns a score to all transcripts at the locus that encode the UniProt canonical isoform. This is the "UniProt score"

5. Length, using two considerations:

    ○ CDS length: The algorithm determines the CDS length of each transcript and then normalises the length with respect to all transcripts at the locus to generate the "Length score".

    ○ Length override: The algorithm determines the CDS length of each transcript and disqualifies any transcript whose CDS length is 75% or less of the longest CDS at the locus. This step aims to avoid known conservation bias towards

shorter isoforms and the override may result in the selected transcript being the one with the second highest (or lower) overall score.

Supplementary Methods 2: Curation Guidelines

As mentioned in the description of the RefSeq Select pipeline in the Supplementary Methods 1, when the Select transcript picked independently by the RefSeq and Ensembl pipelines did not match, or was flagged by QA tests, the structure and sequence of the final select transcript is decided via manual review by EMBL-EBI and RefSeq curators. Since the initiation of the MANE project, the two groups had several discussions and formulated a set of curation guidelines to ensure clear and consistent rules to decide the MANE Select transcript. Some examples of specific scenarios that precluded the choice of the MANE Select transcript by the automated pipelines are provided below. Note: NCBI Genome Data Viewer tracks described in the scenarios below are available in the "Gene Support" track collection in "NCBI recommended tracks" menu under the "Configure" option.

Conservation versus expression

The MANE Select typically contains the longest conserved coding sequence, with the best expressed transcript exon combination. Sometimes, examination of individual exons reveals a discrepancy between the expression levels of the exon versus the conservation of the coding sequence included in it. In such a scenario, conservation of the CDS takes precedence over its expression, in deciding whether to include the exon in the MANE Select or not. An example is *STIM1* (HGNC:11386), where the MANE Select (NM_001382567.1/ ENST00000526596.2)

includes a cassette exon which is not as strongly expressed as the flanking constitutive exons. This is indicated by the recount3 RNA-seq intron-spanning read counts, which favor the exclusion of the exon (1,088,796 reads) over its inclusion (271,888 and 266,684 reads for the two introns, respectively). However, a transcript including this exon was chosen as the MANE Select based on the evolutionary constraint of the CDS seen in PhyloCSF in the 100 vertebrate set and direct evidence of translation from a couple of peptides from the Human Peptide Atlas[42] build 491 (PAp04723979 and PAp04723998).

Incomplete transcript (add 5' UTR exons)

We encountered cases where the transcript chosen as the Select transcript was partial in the 5' UTR region in one or both annotation sets, based on current evidence. For example, although both the RefSeq and the Ensembl pipelines chose matching transcripts for the gene *MAB21L3* (HGNC:26787), QA tests determined that the transcripts in both the annotations may be incomplete at the 5’ end. Upon reviewing archived transcript data from INSDC, CAGE data and the intron-spanning RNA-seq read data from recount3, curators added a 5’ UTR exon to the Select transcripts. The 5’ end of the added exon is based on a CAGE cluster (p1|p4|p3@MAB21L3) that can be seen in the “RefSeq processed FANTOM5 CAGE peaks C, cluster merge threshold: 50nt” track in GDV. The update can be seen in the GDV display of this gene by loading the RefSeq Annotation Release 109_20201120 track, which includes the updated NM_152367.3 and the earlier annotation build, Annotation Release 109_20200522, which shows NM_152367.2 without the extra 5’ UTR exon. The annotation tracks can be added using the ‘Configure’ menu of GDV.

Annotation of the start codon and the 5’ CDS

A QA test that looks for the genomic position of the start codon in the CDS, compared to the location of the transcription start site (TSS), flagged MANE select candidates that had the TSS located downstream of the annotated start codon. Such genes were reviewed by curators, who examined multiple datasets including UCSC multiz alignments, coding sequence conservation in PhyloCSF, peptide data from Human Peptide Atlas and ribosome profiling data from the GWIPS-viz browser[43].

In several cases, the annotated start codon was determined to be wrong based on available data. For example, in the case of the MANE Select candidates for the gene *RNF39* (HGNC:18064) (NM_025236.3 and ENST00000244360.8), the 5'-most start codon in the open reading frame was annotated in the older annotation builds (NCBI Annotation Release 109.20200522 and Ensembl release 102). However, multiz alignments of several mammalian reference genomes (https://tinyurl.com/2p9exe9p) show that: a) the start codon is not conserved in many mammals, b) several mammals harbor indels in the 5' CDS that could potentially disrupt the reading frame. Hence, the two MANE select candidates were updated to begin from the calculated TSS (see Supplementary Methods 3 below for details of the TSS calculation method) and a downstream start codon was annotated.

Similar to the above case, the gene *FLNA* (HGNC:3754) was also flagged for a review of the start codon and the 5' CDS. The principal CAGE cluster p1 aligns to the genomic region corresponding to the second exon of the transcript and downstream of the annotated start codon. However, in contrast to *RNF39*, the start codon and the 5' CDS were found to be conserved based on multiz alignments (https://tinyurl.com/4dk9e26u) as well as PhyloCSF data. Additionally, a significant number of peptides from the human Peptide Atlas provide evidence of the validity of the annotated CDS. This suggested the use of an alternate TSS. RNA-seq data

spanning splice junctions indicate an upstream 5' UTR exon, which is also supported by a CAGE cluster (p3, p4) in the corresponding genomic region. Therefore, the annotated CDS was not changed and the upstream CAGE cluster, which has lesser total tag count compared to the p1 cluster, was used to determine the TSS. This is a departure from the typical scenario, where the strongest CAGE cluster (the cluster with the highest total tag count) is used to determine the TSS and underlines the need for manual review.

Supplementary Methods 3: UTR Algorithm

For the majority of transcripts, the 5' and 3' ends were updated based on CAGE and polyA-seq data through an automated process. Details are provided below.

5' end of transcripts

For 5' ends, we relied on CAGE data from the FANTOM5 project. These data include 2006 runs of CAGE sequenced data on the HelicoScope platform from 1829 distinct samples mapped to the GRCh38 assembly (BioProject: PRJDB1099). We imported the "ctss TotalCounts" data aggregated for all runs, and the CAGE clusters as computed by the FANTOM consortium. Data were imported from

http://fantom.gsc.riken.jp/5/datafiles/reprocessed/hg38_latest/extra/CAGE_peaks/hg38_fair+new_CAGE_peaks_phase1and2.bed.gz and

https://fantom.gsc.riken.jp/5/datahub/hg38/reads/

We further processed the CAGE data to identify CAGE peaks suitable for automatic and manual curation. The overall goal was to pick an exact 5’ end that is biologically valid, representative of the data, and does not result in a transcript that is overly short compared to the available transcript data. We first combined CAGE clusters found in close proximity (within 50 bp of each other) on the same strand. We then re-analyzed the TotalCounts data in the region of each merged cluster to find the maximum peak, and then the 5’-most peak within the merged cluster with a signal within 50% of the max peak, which we referred to as the “longest strong” rule. In many cases this peak is the same as the maximum peak. In others, it selects an upstream transcript end that may not be the absolute strongest single peak but represents a significant fraction of the data and the transcript extension is often supported by additional expression data. The total count of CAGE tags within each merged cluster was also computed. These data are available as a track for viewing and download in NCBI’s Genome Data Viewer (GDV) browser (“RefSeq-processed FANTOM5 CAGE peaks C, cluster merge threshold: 50 bp)” found under the ‘5’ termini support’ category in the ‘Expression’ tab of the browser Configure Page. Individual CAGE clusters and their corresponding longest-strong peaks were matched to individual RefSeq transcripts based on a scan for clusters located either upstream of the existing transcription start site (within 500 bp), or within the first exon. If multiple separate clusters were found within this region, the cluster with the highest overall CAGE counts was chosen. A secondary check for additional upstream CAGE clusters with strong overall counts and a strong peak was also applied and used for manual review. Extensive data review by both RefSeq and EMBL-EBI curators established that the chosen ends were largely compatible with the current curation practices for both groups, and suitable for bulk updates.

To further validate the chosen 5' ends, we used an approach similar to Shamie et al[44] and scanned the genome sequence for several TSS signatures: enrichment of purines (A or G) at the TSS, which is a characteristic of RNA polymerase II transcription initiation, and the presence of TATA box motifs around -30 and/or Inr motifs at +1 relative to the TSS for some transcripts. We compared TSSs for RefSeq Select or Ensembl Canonical transcripts annotated at the beginning of this project and pre-dating bulk CAGE-based updates to what is found in the MANE set and found a pronounced enrichment for purines in the MANE set (88.6%) compared to the initial RefSeq (72.6%) or Ensembl (69.3%) transcripts (Extended Figure 3A). Similarly, MANE transcripts were enriched for Inr motifs positioned at the TSS (1958, compared to 614 or 500 for starting RefSeq and Ensembl, respectively), and TATA boxes positioned in the range of -40 to -10 bp relative to the TSS (corresponding to the core TATAAA motif located between -38 and -18) were found for 2895 MANE transcripts, more than doubling the original datasets (1319 and 1267 for starting RefSeq and Ensembl, respectively) (Extended Figure 3B and 3C). The overall frequency of MANE transcripts with TATA boxes (15.6%) is consistent with prior studies[45,46,47] (<25%). Our analysis of Inr motifs is relatively stringent and requires the best Inr motif in the region to be precisely located at the TSS. Consequently, the observed frequency of MANE transcripts with Inr motifs (10.5%) can't be directly compared to prior studies. The MANE dataset is also restricted to protein-coding genes, and typically uses the strongest promoter for each gene, which may be biased compared to all promoters. Nonetheless, the strong enrichment for TSS signals seen in MANE transcripts compared to the earlier Ensembl and RefSeq datasets demonstrates the value of updating the transcript start sites based on CAGE data.

As with any large-scale dataset, there are some caveats and artifacts introduced by aspects of the CAGE data. The data models and software commonly used with gene annotation sets necessitate

picking a single start and end site for each transcript, even when the underlying biology is much more complex. We have chosen to use data from an aggregate of the entire FANTOM5 dataset, spanning nearly 2000 samples, which will bias our choice of transcript start sites to those that are best represented in those samples. Analyses of specific TSS usage in individual tissues are best done using the original experimental data rather than relying on the specific 5’ ends chosen for MANE transcripts. The FANTOM5 CAGE tags have read lengths of 25-35 bp and were aligned with a non-splice-aware aligner, which will result in failure to identify TSSs where the first exon is less than 25 bp long and will underreport CAGE tags where the first exon is between 25 and 35 bp long. Some short first exons were identified during review of transcripts with weak or no CAGE support, such as IL22 NM_020525.5/ENST00000538666.6 with a 22-nt first exon, but short first exons are likely under-represented within the MANE dataset. Similarly, we postulate that some 5’ UTRs with high GC content and strong secondary structures may result in undersampling of CAGE tags in some regions and selection of a non-optimal TSS. However, the methodology used should select for 5’ ends that represent valid TSSs, even if some are not optimal. In addition, the FANTOM5 CAGE data used for these automated analyses drop tags that map to more than one location in GRCh38, which can result in the partial or complete loss of CAGE tags for some genes, including genes with closely related paralogs. For some genes we manually reviewed additional CAGE data provided by FANTOM5 including multimappers; however, the non-random mapping of the multimapped reads complicate interpretation and make it unsuitable for use in automated curation.

3’ end of transcripts

To define precise 3’ ends, we used published polyA sequencing data generated with a variety of techniques to generate aggregate clusters suitable for defining precise transcript ends. The

original datasets were all mapped relative to GRCh37. To generate suitable data on GRCh38, we aligned several of the largest datasets (SRP041182, SRP003483, SRP007359, SRP133500) (You et al[48], Derti et al[37], Wang et al[31]) using HISAT2[49], pre-processing the reads according to the original publications. For other datasets, we downloaded data from PolyASite[50] for individual runs mapped to GRCh37, and used NCBI Remap[51] to remap the data to GRCh38. The aggregate data was then clustered, filtered for potential artifacts based on genomic polyA content and polyA signal characteristics, and total counts and a preferred polyA site were computed for each cluster based on "longest strong" rules equivalent to those used for 5' ends. The precise location of some polyA sites is ambiguous if found adjacent to one or more A bases in the genome; these sites were shifted to the last A base in the genome such that A bases in the genome are considered part of the terminal exon, corresponding to standard practice for both RefSeq and Ensembl/GENCODE. The computed polyA clusters, along with the polyA site and polyA signal features, are available in the "PolyA sites and clusters" track in GDV.

It's common for transcripts to have multiple polyadenylation sites, in some cases exhibiting tissue-specific regulation. For the majority of the MANE dataset, 3' ends were chosen automatically based on the longest 3' UTR found among curated RefSeq and Ensembl/GENCODE transcripts with the same terminal exon, with the precise 3' end chosen based on the chosen site from the strongest polyA cluster found within 50 bp of the 3' end. This has the effect of including long but rare 3' UTRs in some MANE transcripts, even when the vast majority of transcripts use a more proximal polyA site. For transcripts curated later in the project, we shifted to representing the more typical 3' UTR extensions and omitting long, rare extensions. At this time, we have not re-reviewed 3' UTRs chosen earlier in the project to trim back very rare 3' extensions in favor of expanding the dataset and keeping it stable.

Final transcript end updates

Overall, in the MANE v0.95 dataset, 87.5% of 5' ends are defined by computationally matched CAGE data, 9.6% are manually curated based on transcript and CAGE data, and 2.9% are based on transcript matches between RefSeq and Ensembl in the absence of CAGE data. 84.6% of 3' ends are defined by computationally matched polyA data, 5.6% are manually curated, and 9.9% based on longest transcripts without an associated polyA site from the high-throughput datasets. The need for curation or substitute measures to define transcript ends for 12.5% of 5' ends and 15.4% of 3' ends is a reflection of the depth of data currently available, limitations of mapping strategies especially with closely related paralogs, and low transcription levels for some genes.

Supplementary Methods 4: MANE workflow and quality assurance (QA)

Candidate transcripts for MANE Select were extracted from latest annotations for RefSeq and Ensembl, matched based on splice structures and CDS locations, evaluated for suitability for MANE Select using a set of QA metrics, and updated in batch if all QA metrics passed and suitable data for updates was available.

The RefSeq and Ensembl genome annotations run on different release cycles; to incorporate update steps to synchronize sets of transcripts in both datasets, we adopted a workflow where candidate MANE Select transcripts in RefSeq were identified and updated to refine transcript ends and match the reference genome sequence. The RefSeq genome annotation was then

updated, and the data was subsequently used to refine the transcript ends of equivalent Ensembl transcripts early in the Ensembl build release cycle. The MANE release process was repeated on a 3-month cycle, with public MANE releases typically available when the RefSeq transcripts are public and annotated in their final state, but before the corresponding Ensembl release was public, in order to expedite growth of the dataset.

The basic dataflow was constructed in a SQL database at NCBI, using a Jupyter notebook with C++ and Python code to load supporting data and a series of SQL stored procedures to evaluate the data. The RefSeq transcripts available in each annotation release were compared to pre-release data from Ensembl to identify those Ensembl transcripts with identical splice sites, the same number of exons, and the same start and stop codons, but allowing for length differences in the 5' and 3' UTR exons. In rare cases where multiple matching transcripts were found, a best pick was made preferring GENCODE-curated transcripts and length similarity. Within the RefSeq and Ensembl annotation sets, candidates for Select chosen by each pipeline were identified and compared; if equivalent transcripts were chosen, this became a candidate for MANE Select subject to additional QA.

An extensive set of QA metrics was devised to assess whether a transcript was suitable for automatic selection as MANE Select and for automated updates of the transcript ends, or if curator review was required. Individual QA tests were categorized to block either automatic choice as MANE Select or UTR updates, or were provided to help with curation (Supplementary Table 1, provided as an Excel spreadsheet). A system to override QA tests was devised using either automated criteria, or curator review. For example, the RefSeq and Ensembl pipelines may have both picked the same transcript as Select; however, if another transcript was available using a much stronger promoter, that would trigger the 'not_best_cage3x' test prompting curator

review to see if a transcript using the stronger promoter was a better choice. In general, the QA tests were designed to identify genes in need of curation in either or both of the RefSeq and Ensembl datasets, in particular for cases where transcripts ideal for Select may be missing. For example, some candidate transcripts had an incompletely defined 5' structure and needed to be extended to connect to a promoter; or curated transcripts incorporating as many conserved exons as biologically possible might not have been available in current datasets.

Once an equivalent pair of RefSeq and Ensembl transcripts were identified as Select candidates and any QA tests were resolved, data within the RefSeq database was updated to revise the UTRs based on the computed ends, the revised RefSeq transcript was updated in the public NCBI nucleotide database, and the revised transcript was incorporated into the annotated RefSeq genome. Concurrently, equivalent updates were made to the matching Ensembl transcripts and genome annotation. After updates, the revised annotations were reprocessed through the MANE workflow to ensure that all QA issues were fully resolved, and that the transcript and CDS annotations were identical. The pairs of transcript and protein sequences were also compared. To qualify for inclusion in a MANE release, pairs of RefSeq and Ensembl transcripts must have exactly matching exon structures, CDSs, and identical transcript and protein sequences.

For each MANE release, we provided a set of files on FTP including GFF3 and GTF genome annotation files and RNA and protein FASTA files using either RefSeq or Ensembl identifiers. We also provided a summary report listing the full set of MANE transcripts in the release with a full set of gene, transcript, and protein identifiers. In addition, bigBed files were generated and provided as a track hub for use in different genome browsers. The FTP site is provided with symlinks to provide a stable path to current data, as well as access to previous MANE releases. See Extended Data Table 1 for links to all our data.

Supplementary References

42. Desiere, F. *et al.* The PeptideAtlas project. *Nucleic Acids Res.* **34**, D655–8 (2006).
43. Kiniry, S. J., Michel, A. M. & Baranov, P. V. The GWIPS-viz Browser. *Curr. Protoc. Bioinformatics* **62**, e50 (2018).
44. Shamie, I. *et al.* A Chinese hamster transcription start site atlas that enables targeted editing of CHO cells. *bioRxiv* 2020.10.09.334045 (2020) doi:10.1101/2020.10.09.334045.
45. Gershenzon, N. I. & Ioshikhes, I. P. Synergy of human Pol II core promoter elements revealed by statistical sequence analysis. *Bioinformatics* **21**, 1295–1300 (2005).
46. Carninci, P. *et al.* Genome-wide analysis of mammalian promoter architecture and evolution. *Nat. Genet.* **38**, 626–635 (2006).
47. Yang, C., Bolotin, E., Jiang, T., Sladek, F. M. & Martinez, E. Prevalence of the initiator over the TATA box in human and yeast genes and identification of DNA motifs enriched in human TATA-less core promoters. *Gene* **389**, 52–65 (2007).
48. You, L. *et al.* APASdb: a database describing alternative poly(A) sites and selection of heterogeneous cleavage sites downstream of poly(A) signals. *Nucleic Acids Res.* **43**, D59–67 (2015).
49. Kim, D., Paggi, J. M., Park, C., Bennett, C. & Salzberg, S. L. Graph-based genome alignment and genotyping with HISAT2 and HISAT-genotype. *Nat. Biotechnol.* **37**, 907–915 (2019).
50. Herrmann, C. J. *et al.* PolyASite 2.0: a consolidated atlas of polyadenylation sites from 3' end sequencing. *Nucleic Acids Res.* **48**, D174–D179 (2020).

51. Kitts, P. A. *et al.* Assembly: a resource for assembled genomes at NCBI. *Nucleic Acids Res.* **44**, D73–80 (2016).
