# Supplementary material for: A joint NCBI and EMBL-EBI transcript set for clinical genomics and research
Source: Nature. 2022 Apr 6;604(7905):310–5. doi: 10.1038/s41586-022-04558-8 (PMC9007741; doi:10.1038/s41586-022-04558-8)
Supplement: Supplementary file 2 — Reporting Summary [file 41586_2022_4558_MOESM2_ESM.pdf]

nature portfolio

Corresponding author(s): Dr. Terence Murphy

Last updated by author(s): 12/15/2021

Reporting Summary

Nature Portfolio wishes to improve the reproducibility of the work that we publish. This form provides structure for consistency and transparency in reporting. For further information on Nature Portfolio policies, see our Editorial Policies and the Editorial Policy Checklist.

Statistics

For all statistical analyses, confirm that the following items are present in the figure legend, table legend, main text, or Methods section.

n/a	Confirmed	
☒	☐	The exact sample size (*n*) for each experimental group/condition, given as a discrete number and unit of measurement
☒	☐	A statement on whether measurements were taken from distinct samples or whether the same sample was measured repeatedly
☒	☐	The statistical test(s) used AND whether they are one- or two-sided<br>*Only common tests should be described solely by name; describe more complex techniques in the Methods section.*
☒	☐	A description of all covariates tested
☒	☐	A description of any assumptions or corrections, such as tests of normality and adjustment for multiple comparisons
☒	☐	A full description of the statistical parameters including central tendency (e.g. means) or other basic estimates (e.g. regression coefficient) AND variation (e.g. standard deviation) or associated estimates of uncertainty (e.g. confidence intervals)
☒	☐	For null hypothesis testing, the test statistic (e.g. *F*, *t*, *r*) with confidence intervals, effect sizes, degrees of freedom and *P* value noted<br>*Give P values as exact values whenever suitable.*
☒	☐	For Bayesian analysis, information on the choice of priors and Markov chain Monte Carlo settings
☒	☐	For hierarchical and complex designs, identification of the appropriate level for tests and full reporting of outcomes
☒	☐	Estimates of effect sizes (e.g. Cohen's *d*, Pearson's *r*), indicating how they were calculated

Our web collection on statistics for biologists contains articles on many of the points above.

Software and code

Policy information about availability of computer code

Data collection	No software was used for data collection
Data analysis	The analysis code used for this study is largely integral to the RefSeq and Ensembl/GENCODE curation databases, and not designed for use in isolation or on other annotation datasets. The most critical aspect of the analysis is from tens of thousands of person-hours in curator review to produce the final dataset. HOMER v4.11 is available from http://homer.ucsd.edu/homer/. FIMO v5.3.2 is available from https://meme-suite.org/meme/meme_5.3.2/doc/fimo.html. HISAT 2.2.1 is available from http://daehwankimlab.github.io/hisat2/.

For manuscripts utilizing custom algorithms or software that are central to the research but not yet described in published literature, software must be made available to editors and reviewers. We strongly encourage code deposition in a community repository (e.g. GitHub). See the Nature Portfolio guidelines for submitting code & software for further information.

Data

Policy information about availability of data

All manuscripts must include a data availability statement. This statement should provide the following information, where applicable:

- Accession codes, unique identifiers, or web links for publicly available datasets
- A description of any restrictions on data availability
- For clinical datasets or third party data, please ensure that the statement adheres to our policy

The datasets generated during the current study are available from NCBI's FTP site (https://ftp.ncbi.nlm.nih.gov/refseq/MANE/MANE_human/) and can also be accessed from EMBL-EBI's Transcript Archive (Tark) page (http://tark.ensembl.org/web/mane_project/).

The datasets analyzed during the current study can be accessed using the following resources:

Ensembl/GENCODE annotation: All Ensembl/GENCODE annotation builds used in the comparison of RefSeq and Ensembl/GENCODE transcripts for determining transcript matches in MANE analysis are available in release 96-105 directories on the Ensembl FTP site (e.g. http://ftp.ensembl.org/pub/release-105/gtf/homo_sapiens/Homo_sapiens.GRCh38.105.gtf.gz)

RefSeq Annotation: All RefSeq annotation builds used in the comparison of RefSeq and Ensembl/GENCODE transcripts for determining transcript matches in MANE analysis are available at https://ftp.ncbi.nlm.nih.gov/genomes/refseq/vertebrate_mammalian/Homo_sapiens/annotation_releases/

Ensembl Canonical Transcripts: The Ensembl canonical transcripts used for the comparison between gnomAD vs ClinVar vs MANE were from Ensembl release 103. These can be accessed using the Ensembl Perl API for release 103 using this call on the gene: http://www.ensembl.org/info/docs/Doxygen/core-api/classBio_1_1EnsEMBL_1_1Gene.html
Alternatively, the same data are available via the Ensembl REST API, using the lookup endpoint: https://jan2020.rest.ensembl.org/documentation/info/lookup.

CAGE: Aggregated "CTSS TotalCounts" CAGE data and the CAGE clusters as computed by the FANTOM consortium was imported from http://fantom.gsc.riken.jp/5/datafiles/reprocessed/hg38_latest/extra/CAGE_peaks/hg38_fair+new_CAGE_peaks_phase1and2.bed.gz and https://fantom.gsc.riken.jp/5/datahub/hg38/reads/

Poly A: PolyA-seq data used to generate polyA clusters and determine polyA sites were from multiple studies listed in references 31-37. The data are available in study accessions SRP041182, SRP003483, SRP007359, SRP133500 in NCBI's Sequence Read Archive (SRA: https://www.ncbi.nlm.nih.gov/sra) and from PolyASite 2.0 (https://www.polyasite.unibas.ch/).

APPRIS: APPRIS data is available at https://appris.bioinfo.cnio.es/#/downloads. It is updated for every Ensembl/GENCODE release. APPRIS data is based on Ensembl releases 95 - 104.

PhyloCSF: PhyloCSF data used to identify conserved sequences were imported from https://data.broadinstitute.org/compbio1/PhyloCSFtracks/.

Recount3: Intron support data from Snaptron/recount3 was imported from http://snaptron.cs.jhu.edu/data/.

Field-specific reporting

Please select the one below that is the best fit for your research. If you are not sure, read the appropriate sections before making your selection.

☒ Life sciences ☐ Behavioural & social sciences ☐ Ecological, evolutionary & environmental sciences

For a reference copy of the document with all sections, see nature.com/documents/nr-reporting-summary-flat.pdf

Life sciences study design

All studies must disclose on these points even when the disclosure is negative.

Sample size	No experiments where sample size is relevant were conducted for this study
Data exclusions	No experiments where data exclusion is applicable were conducted for this study
Replication	No experiments where replication is applicable were conducted for this study
Randomization	No experiments where randomization is applicable were conducted for this study
Blinding	No experiments where blinding is applicable were conducted for this study

Reporting for specific materials, systems and methods

We require information from authors about some types of materials, experimental systems and methods used in many studies. Here, indicate whether each material, system or method listed is relevant to your study. If you are not sure if a list item applies to your research, read the appropriate section before selecting a response.

Materials & experimental systems

n/a	Involved in the study	
☒	☐	Antibodies
☒	☐	Eukaryotic cell lines
☒	☐	Palaeontology and archaeology
☒	☐	Animals and other organisms
☒	☐	Human research participants
☒	☐	Clinical data
☒	☐	Dual use research of concern

Methods

n/a	Involved in the study	
☒	☐	ChIP-seq
☒	☐	Flow cytometry
☒	☐	MRI-based neuroimaging
